# Contributing factors to advanced brain aging in depression and anxiety disorders

**DOI:** 10.1038/s41398-021-01524-2

**Published:** 2021-07-21

**Authors:** Laura K. M. Han, Hugo G. Schnack, Rachel M. Brouwer, Dick J. Veltman, Nic J. A. van der Wee, Marie-José van Tol, Moji Aghajani, Brenda W. J. H. Penninx

**Affiliations:** 1 Department of Psychiatry, Amsterdam University Medical Centers, Vrije Universiteit and GGZ inGeest, Amsterdam Neuroscience, Amsterdam, The Netherlands; 2 Department of Psychiatry, UMCU Brain Center, University Medical Center Utrecht, Utrecht, Netherlands; 3 Department of Complex Trait Genetics, Centre for Neurogenomics and Cognitive Research, VU University, Amsterdam, The Netherlands; 4 Leiden Institute for Brain and Cognition, Leiden University, Leiden, The Netherlands; 5 Department of Psychiatry, University Medical Center Leiden, Leiden, The Netherlands; 6 Cognitive Neuroscience Center, University Medical Center Groningen, University of Groningen, Groningen, The Netherlands; 7 Institute of Education & Child Studies, Section Forensic Family & Youth Care, Leiden University, Leiden, The Netherlands

**Keywords:** Neuroscience, Depression

## Abstract

Depression and anxiety are common and often comorbid mental health disorders that represent risk factors for aging-related conditions. Brain aging has shown to be more advanced in patients with major depressive disorder (MDD). Here, we extend prior work by investigating multivariate brain aging in patients with MDD, anxiety disorders, or both, and examine which factors contribute to older-appearing brains. Adults aged 18–57 years from the Netherlands Study of Depression and Anxiety underwent structural MRI. A pretrained brain-age prediction model based on >2000 samples from the ENIGMA consortium was applied to obtain brain-predicted age differences (brain PAD, predicted brain age minus chronological age) in 65 controls and 220 patients with current MDD and/or anxiety. Brain-PAD estimates were associated with clinical, somatic, lifestyle, and biological factors. After correcting for antidepressant use, brain PAD was significantly higher in MDD (+2.78 years, Cohen’s *d* = 0.25, 95% CI −0.10-0.60) and anxiety patients (+2.91 years, Cohen’s *d* = 0.27, 95% CI −0.08-0.61), compared with controls. There were no significant associations with lifestyle or biological stress systems. A multivariable model indicated unique contributions of higher severity of somatic depression symptoms (*b* = 4.21 years per unit increase on average sum score) and antidepressant use (−2.53 years) to brain PAD. Advanced brain aging in patients with MDD and anxiety was most strongly associated with somatic depressive symptomatology. We also present clinically relevant evidence for a potential neuroprotective antidepressant effect on the brain-PAD metric that requires follow-up in future research.

## Introduction

Depression and anxiety are common and often comorbid mental health disorders, and their effects can broadly impact a person’s life. There is a plethora of evidence showing poorer quality of life, functional disability, and increased mortality burden in these patients [1, 2]. Depression and anxiety disorders further represent a risk factor for aging-related conditions [3–5], as studies show consistent evidence for poorer somatic and chronic disease profiles in these patient groups [6], often with a premature onset. Importantly, the incidence and burden of these disorders are a strain on society, which has an important challenge to face in the coming years, as the number of people aged >65 is expected to reach 1.6 billion in 2050 [7]. Advancing mental health and well-being across the lifespan and into old age should, therefore, be a major priority on the research agenda.

Multivariate pattern-recognition techniques, and especially machine-learning methods, have promoted a steep increase in the development of ways to measure and quantify aging [8]. Central to this field is that multivariate (biological) patterns are utilized and integrated into a single score: the biological age. Biological age can be derived from, for instance, omics data (e.g., epigenetic clocks), but also clinical biomarkers obtained from, for example, blood chemistries [9]. In the current study, we focus on biological age based on a validated method of MRI-derived brain structure [10, 11], with brain-predicted age difference (brain PAD, predicted brain age minus chronological age) [12] as the main outcome. This metric is relative to one’s chronological age, such that positive values indicate an older-appearing brain, and negative values resemble a younger-appearing brain than normally expected at that age.

A handful of studies have investigated brain PAD in depression, with studies showing +4.0 years [13], as well as no significantly increased brain age [14, 15]. Recent findings from the Enhancing NeuroImaging Genetics through Meta-Analysis (ENIGMA) consortium using a more than ten-fold larger pooled sample of MDD patients than the largest previous study suggest a 1.1-year higher brain-PAD in MDD patients as compared with controls [16]. However, this difference did not seem to be driven by specific clinical characteristics (recurrent status, remission status, antidepressant medication use, age of onset, or symptom severity). An important aspect that remains relatively unknown is which underlying mechanisms cause the brain-age metric to advance in depression, and, despite the increase of brain-age studies in the past decade, in general [11].

Large pooled datasets from global consortia offer the statistical power needed to detect small-effect sizes usually observed in MDD, but a limitation of consortium data is that its collection is commonly not harmonized across all sites and cohorts. Here, we underline the complementary value of a more homogeneous and clinically well-characterized sample from the Netherlands Study of Depression and Anxiety (NESDA), to gain more insight into the observed brain PAD difference between MDD patients and controls. We extend prior work by exploring which specific symptom clusters (mood/cognition, immunometabolic, and somatic) of MDD are associated with brain PAD. To the best of our knowledge, there are currently no brain-age studies in anxiety disorders, although higher brain PAD has been observed in post-traumatic stress disorder [17]. Given the frequent co-occurrence and correlated symptoms [18] of depression and anxiety (i.e., family of internalizing disorders) [19], we also extend prior work by including patients with MDD and/or anxiety disorders in the current study.

Evidence is starting to emerge that brain PAD is associated with reduced mental and somatic health, such as with stroke history, diabetes diagnosis, smoking, alcohol consumption, and some cognitive measures [20], but also intrinsic measures such as genetic variants [21, 22]. This study seeks to further address the research gaps, by examining whether three commonly dysregulated biological stress systems in depression and anxiety disorders (inflammation, hypothalamic pituitary adrenal [HPA] axis, autonomic nervous system [ANS]) were predictive of brain aging. Disruptions and dysregulations in these stress systems were hypothesized to result in advanced brain aging across diagnostic groups. We further associated various clinical, lifestyle, and somatic health indicators with the brain-PAD metric for our primary hypothesis to identify unique contributing factors to brain aging.

## Materials and methods

### Study sample

A subsample of subjects of the NESDA were included for the MRI substudy (total *N* = 301). Twelve participants were excluded due to poor image quality, two because of claustrophobia, one control subject due to high depression rating (Montgomery Asberg Depression Rating Scale score >8), and one due to the large time difference between the psychiatric and biological and MRI measurements (total excluded, *N* = 16). For the current study, we therefore included *N* = 65 controls (65% female, aged 21–55) and *N* = 220 patients with a current depressive and/or anxiety disorder (69% female, aged 18–57). The NESDA sample consisted of more than 94% persons from North European origin. The current study was approved by the ethical review boards of the three participating centers (Amsterdam, Groningen, and Leiden) and informed consent of all participants was obtained.

### Image processing and analysis

Magnetic resonance imaging (MRI) data were obtained using three independent 3 T Philips MRI scanners (Philips Healthcare, Best, The Netherlands) located at different participating centers. Scanners were equipped with a SENSE 8-channel (Leiden University Medical Center and University Medical Center Groningen) and a SENSE 6-channel (Academic Medical Center) receiver head coil (Philips Healthcare). Standardized image segmentation and feature-extraction protocols, using the FreeSurfer processing software, developed by the ENIGMA consortium were used (http://enigma.ini.usc.edu/protocols/imaging-protocols/) to extract 153 features from regions of interest, including the volumes of 14 subcortical gray matter regions (bilateral nucleus accumbens, amygdala, caudate, hippocampus, pallidum, putamen, and thalamus) and the two lateral ventricles, cortical thickness and surface area from 68 cortical regions, and total intracranial volume (ICV). Visual inspection of the segmentations showed that the pallidum was underestimated in 27 individuals, and poor segmentations of the thalamus (*N* = 1), caudate (*N* = 2), putamen (*N* = 9), and accumbens (*N* = 1) were observed. These individual features were subsequently median-imputed. In addition, segmentations were statistically examined for outliers and the FreeSurfer feature was excluded if it was >2.698 standard deviations away from the global mean. However, if a sample was a statistical outlier, but visual inspection showed that it was properly segmented, it was kept in the dataset (0.4% of features).

### FreeSurfer brain age prediction model

We used a publicly available brain age model (https://www.photon-ai.com/enigma_brainage/) that was trained to predict age from 77 ((left+right hemisphere features)/2 and ICV) FreeSurfer features (for more detail, see [16]). Briefly, the ridge regression coefficients learned from two separate models trained on 952 male and 1236 female control subjects (aged 18–75 years), respectively, were directly applied to the features of the current samples (*N* = 285), also separately in male and female groups. Of note, NESDA was not part of the development of the ENIGMA model, and the current dataset is thus completely independent. The model’s generalization performance was assessed by calculating several metrics: (a) the correlation between predicted brain age and chronological age, (b) the amount of chronological age variance explained by the model (R^2^), (c) the mean absolute error (MAE) between predicted brain age and chronological age, and (d) root mean squared error (RMSE).

### Diagnostic ascertainment

Participants in the current study included control subjects (no lifetime history of psychiatric disorders) and patients with a current depression and/or current anxiety disorder (i.e., generalized anxiety disorder, panic disorder, and social anxiety disorder) within a 6-month recency. The Composite International Diagnostic Interview (CIDI version 2.1) was used as a diagnostic instrument to ascertainment [23].

### Clinical assessment

We examined several clinical variables as predictors, including (a) depressive symptoms as measured by the summary score of the Inventory for Depressive Symptoms (IDS) at the time of scanning [24], (b) anxiety symptoms as measured by the summary score of the Beck Anxiety Inventory (BAI) at the time of scanning [25], (c) cumulative childhood trauma index [26] (before the age of 16) as measured by a childhood trauma interview, and (d) recent negative life events in the past year as measured with the Brugha questionnaire [27]. Depressive symptoms from the IDS were also categorized into three separate clusters (mood/cognition, somatic, and immunometabolic symptoms). The mood/cognition and somatic symptom clusters were based on findings from a principal component analysis (PCA) on a larger sample of ~3000 individuals [28], but a separate factor indicating an immuno-metabolic symptom profile was added [29]. Briefly, the mood/cognition cluster consisted of 16 items (e.g., feeling sad, irritable, anxious or tense, concentration/decision-making problems, and general interest/interest in sex), the immunometabolic cluster consisted of five atypical/energy-related items (i.e., sleeping too much, increased appetite, increased weight, low-energy level/fatigue, and leaden paralysis), and the somatic cluster consisted of 13 items concerning bodily problems (e.g., sleeping problems, aches and pains, and constipation/diarrhea). Within the patients only, we also investigated associations with: (a) duration of symptoms, (b) age of onset of illness, and (c) antidepressant medication use selective serotonin-reuptake inhibitors (ATC code N06AB) and other antidepressants (ATC codes N06AF, N06AG, N06AX). See Supplement for more details on these measures and Supplementary Table S1 for a complete overview of the depression-symptom profiles.

### Somatic health assessment

Body mass index (BMI) was assessed during an interview by dividing a person’s weight (in kilogram [kg]) by the square of their height (in meter [m]). The number of self-reported current somatic diseases (heart disease, epilepsy, diabetes, osteoarthritis, cancer, stroke, intestinal disorders, ulcers, and lung-, liver-, and thyroid disease) for which participants received medical treatment was counted.

### Lifestyle assessment

Smoking status was expressed by calculating the number of cigarettes smoked per day. Alcohol consumption was expressed as the mean number of drinks consumed per week, measured by the AUDIT [30]. Physical activity was assessed using the International Physical Activity Questionnaire (IPAQ) and expressed in total metabolic equivalent (MET) minutes per week [31].

### Biological stress assessment

We included predictors from three major biological stress systems: (a) the immune-inflammatory system (C-reactive protein [CRP], interleukin-6 (IL6), and tumor necrosis factor-α (TNF-ɑ)), (b) the hypothalamic pituitary adrenal (HPA) axis (cortisol-awakening response [CAR] and evening cortisol), and (c) the autonomic nervous system (ANS: heart rate, respiratory sinus arrhythmia [RSA], and pre-ejection period [PEP]). Details can be found in Supplement.

### Statistical analysis

All statistical analyses were performed using R version 3.5.3 (R Core Team, 2019). To confirm and extend the findings from earlier work [16], we first used linear regressions to examine brain PAD differences between the control and patient groups and explored brain-PAD associations with several clinical characteristics within the patients only (i.e., duration of symptoms, age of onset of illness, and AD use). Second, we used separate linear regression models with brain PAD as measured outcome and variables of interest as a predictor to explore and select contributors in all participants, irrespective of the diagnostic group. Finally, to test our primary aim, stepwise regression with forward selection was used to successively add significant contributors (uncorrected for multiple comparisons) to an intercept-only model, starting with the variable that explained most variance and stopping if the model fit did not improve anymore. The best subset of variables leading to the best model fit (i.e., lowest Akaike’s Information Criterion [AIC]) was selected to examine unique contributions to brain PAD. To test the robustness of the findings, we also repeated the stepwise regression using a backward elimination procedure, starting with all predictors in the model, and iteratively removing the least contributing predictors, and stopping when the model only includes statistically significant predictors. Given the richness of the NESDA dataset, we additionally computed exploratory intercorrelations between the brain-PAD metric and other available biological age indicators (i.e., telomere length, epigenetic, transcriptomic, proteomic, and metabolomic age) in an overlapping sample of *N* = 98, while correcting for chronological age. Inflammatory predictors were log_e_-transformed due to highly skewed distributions and subsequently corrected for fasting status and anti-inflammatory medication use. ANS predictors were corrected for fasting status, heart medication use, and mean arterial blood pressure. HPA predictors were corrected for fasting status, awakening time, variable indicating whether it was a working day or not, season, and smoking. All biological stress markers >3*sd away from the mean were winsorized. Brain-PAD (predicted brain age minus age) was used as the outcome, and age, sex, education level (years), and two dummy variables for scan location were included as predictor variables in all models. Analyses were tested two-sided and findings were considered statistically significant at *p* < 0.05. All *b-*regression coefficients from all models may be interpreted as added brain aging in years in response to each unit increase of the predictor.

## Results

### Sample characteristics

Demographics and assessed phenotypes of the current study sample can be found in Table 1. Briefly, the patient group consisted of patients with a current MDD diagnosis but no anxiety (28.2%), patients with a current anxiety disorder but no depression (30.5%), and patients with a current comorbid depression and anxiety disorder (41.4%). The patient group (mean 37.37 ± SD 10.20 years) was younger than the control group (mean 40.81 ± SD 9.78 years) and had fewer years of education (mean 14.28 ± SD 2.86 years in controls vs. mean 12.39 ± SD 3.19 in patients). Control and patient groups were similar in terms of male/female ratios, but not distributed equally between scan locations (Amsterdam, Leiden, and Groningen) (Χ_(2)_=6.26, *p* = 0.044).

**Table 1:** Participant characteristics of controls and patients.

Characteristic	*N*^a^	Controls, *N* = 65^b^	Patients, *N* = 220^b^	*p*-value^c^
*Demographics*				
Age (years)	285	40.81 ± 9.78 (21.26–56.67)	37.37 ± 10.20 (17.76–57.17)	**0.02**
Female Sex	285	42 (65%)	152 (69%)	0.60
Education Level (years)	285	14.28 ± 2.86 (5.00–18.00)	12.39 ± 3.19 (5.00–18.00)	**<0.001**
Scan location	285			**0.04**
1		26 (40%)	66 (30%)	
2		27 (42%)	78 (35%)	
3		12 (18%)	76 (35%)	
*Clinical characteristics*				
Major depressive disorder			62 (28%)	
Anxiety disorder			67 (30%)	
Comorbid depression and anxiety			91 (41%)	
Total depression severity score	280	4 ± 4 (0–21)	23 ± 12 (1–57)	**<0.001**
Total anxiety severity score	278	2 ± 3 (0–11)	14 ± 10 (0–50)	**<0.001**
Mood/cognition symptom cluster	285	1.09 ± 0.13 (1.00–1.47)	1.86 ± 0.50 (1.00–3.27)	**<0.001**
Somatic depression symptom cluster	285	1.20 ± 0.21 (0.90–2.20)	1.64 ± 0.41 (0.80–2.80)	**<0.001**
Immunometabolic symptom cluster	285	1.11 ± 0.21 (0.80–1.80)	1.60 ± 0.48 (0.60–3.60)	**<0.001**
Childhood Trauma Index	285	1 ± 1 (0–8)	2 ± 2 (0–8)	**<0.001**
Recent negative life events	285	0.57 ± 0.83 (0.00–3.00)	0.89 ± 1.09 (0.00–3.00)	0.05
*Within patients*				
Antidepressant use	220		77 (35%)	
Duration of depressive symptoms (proportion of time in the past 4 years)	190		0.34 ± 0.28 (0.00–1.00)	
Duration of anxiety symptoms (proportion of time in the past 4 years)	192		0.42 ± 0.35 (0.00–1.00)	
Age of onset of depression (years)	191		23.75 ± 10.44 (4.00–54.00)	
Age of onset of anxiety (years)	170		18.15 ± 10.93 (4.00–52.00)	
*Somatic health*				
Body Mass Index (kg/m^2^)	285	24.36 ± 3.73 (19.03–37.42)	25.14 ± 4.72 (18.04–42.21)	0.35
Number of somatic diseases	285	0 ± 1 (0–3)	0 ± 1 (0–3)	0.66
*Lifestyle*				
Alcohol intake (mean number of drinks per week)	285	6.2 ± 6.1 (0.0–25.0)	4.3 ± 6.5 (0.0–47.5)	**0.01**
Smoking behavior (cigarettes/day)	161	9.26 ± 7.61 (0.00–29.00)	12.56 ± 10.73 (0.00–70.00)	0.09
Physical activity (1,000 MET minutes per week)	271	3.8 ± 3.3 (0.3–16.5)	3.6 ± 3.5 (0.0–17.1)	0.19
*Inflammation*				
C-Reactive Protein (mg/l)	280	−0.03 ± 0.52 (−1.00–1.08)	0.11 ± 0.59 (−1.00–1.35)	0.17
Tumor Necrosis Factor-α (pg/ml)	279	−0.16 ± 0.26 (−1.00–0.63)	−0.12 ± 0.26 (−1.00–0.63)	0.39
Interleukin-6 (pg/ml)	280	−0.15 ± 0.31 (−1.12–0.57)	−0.14 ± 0.48 (−2.29–1.74)	0.89
*Autonomic Nervous System*				
Resting Heart Rate (bpm)	276	69 ± 8 (51–86)	68 ± 10 (44–96)	0.62
Respiratory Sinus Arrhythmia (ms)	276	51 ± 25 (14–130)	49 ± 26 (7–130)	0.59
Pre-injection Period (ms)	276	119 ± 17 (81–147)	119 ± 16 (75–168)	0.66
*HPA-axis*				
Cortisol Awakening Response Area under the curve with respect to the ground (nmol/l/hr)	197	0.57 ± 4.63 (−13.06–11.13)	2.44 ± 5.74 (−14.92–19.00)	0.10
Cortisol Awakening Response Area under the curve with respect to the increase (nmol/l/hr)	197	16.89 ± 4.88 (8.49–32.13)	18.51 ± 6.75 (5.36–37.97)	0.23
Evening cortisol (nmol/l)	207	5.05 ± 2.44 (2.12–13.33)	5.04 ± 2.43 (1.09–12.96)	0.90

Bold p-values indicate significance at the p < 0.05 level

^a^N indicates non-missing observations

^b^Statistics presented: mean ± SD (minimum–maximum); *n* (%)

^c^Statistical tests performed: Wilcoxon rank-sum test; chi-square test of independence.

### Brain age prediction performance

**Fig. 1:**
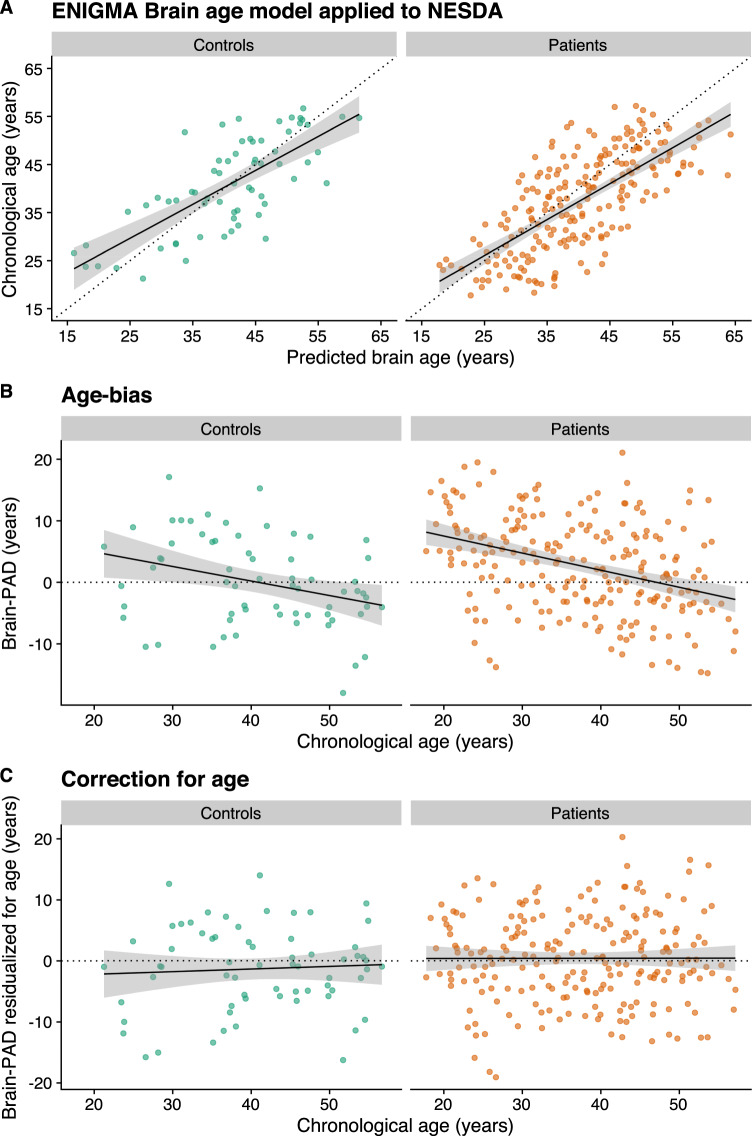
Brain-age prediction. **A** Correlation between predicted brain age and chronological age in controls (*r* = 0.73, *R*^2^ = 0.45, *p* < 0.0001) and patients (*r* = 0.72, *R*^2^ = 0.36, *p* < 0.0001). Of note, predicted brain age reflects estimates corrected for the offset (brain age_corrected_ = brain age − brain PAD − mean brain-PAD_controls_). **B** There was a residual effect of age on the brain-PAD outcome in controls (*r* = −0.32, *p* = 0.01) and patients (*r* = −0.37, *p* < 0.0001), **C** which was statistically corrected for by adding age as a covariate in all models.

Site-specific and other heterogeneous sources of variation challenge the external generalization of machine learning models in general, and brain age prediction models in specific. Using the ENIGMA brain age model (www.photon-ai.com/enigma_brainage), we obtained a correlation of *r* = 0.73 in the control subjects and *r* = 0.72 in the patient group between predicted and chronological age, but in both groups, brain-age predictions were overestimated (mean brain PAD [SD], 8.18 [7.27] years in controls and 10.86 [7.73] years in patients). The current sample showed significantly lower cortical thickness values compared with healthy control samples from the same age range obtained from other scanners, specifically those that were used to train the brain-age prediction model [16]. Given that the ENIGMA brain age model mostly relies on thickness features to make predictions, and since thickness decreases with age, the brain ages of the NESDA participants were consistently overestimated by the model. To correct for the offset, we calculated the mean brain PAD in the control group and subtracted this from all individual brain-PAD estimates. This correction resulted in an R^2^ of 0.45 and MAE of 5.97 (SD 4.09) years in controls, and R^2^ of 0.36 and MAE of 6.73 (4.64) years in patients. Important to note, however, is that we only subtracted a constant from each individual brain-age prediction in both controls and patients. While such a linear operation results in different group mean values, it does not change the relative values of brain age and brain PAD between individuals, and therefore has no effect on subsequent statistics, including group comparisons and associations. Figure 1A shows the unaffected correlation between predicted brain age (*x* axis) and chronological age (*y* axis) in control subjects (*r* = 0.73, *p* < 0.0001) and in patients (*r* = 0.72, *p* < 0.0001). There also was also a well-known and commonly described age bias (i.e., correlation between brain-PAD and age) [17, 32, 33] in controls (*r* = −0.32, *p* = 0.01) and patients (*r* = −0.37, *p* < 0.0001) in the current sample (Fig. 1B), which was statistically dealt with by including age as a predictor variable in further analyses (Fig. 1C) [32]. While other bias-correction procedures exist that explicitly correct the predictions or brain-PAD metric, adding age as a covariate in subsequent analyses is equally effective [34]. In contrast to the ENIGMA analyses, the goodness of fit did not further improve by adding a quadratic age term (*F* = 0.28, *p* = 0.60), and, therefore, only a linear-age term was included.

### Advanced brain aging in depression and anxiety disorders

**Fig. 2:**
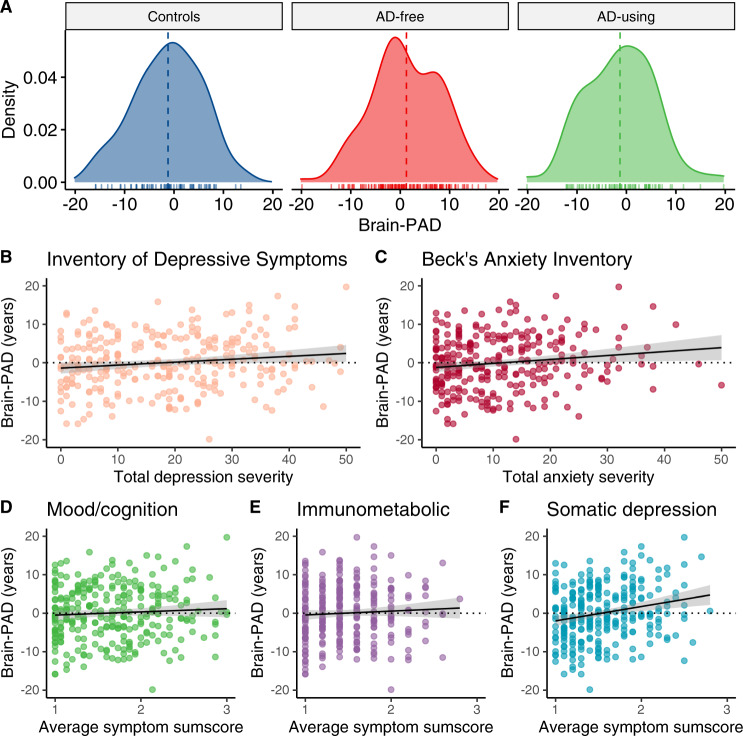
Brain-PAD differences and clinical characteristics. A AD-free patients showed significantly higher brain PAD compared with AD-using patients (+2.58 years [SE 1.02 years], Cohen’s *d* = 0.36, 95% CI 0.09–0.64) and controls (+2.63 years [SE 1.10 years], Cohen’s *d* = 0.31, 95% CI 0.01–0.60). **B** Advanced brain aging was associated with overall higher total depressive symptoms (*b* = 0.07 years per unit increase on the Inventory of Depressive Symptoms, *p* = 0.03), **C** total anxiety symptoms (*b* = 0.11 years per unit increase on the Beck’s Anxiety Inventory, *p* = 0.01), but not specifically with **D** the mood/cognition (*b* = 0.89 years per unit increase on average sum score, *p* = 0.27) or **E** immunometabolic (*b* = 0.45 years per unit increase on the average sum score, *p* = 0.62) symptom cluster. The association in (**B**) seemed to be driven mostly by **F** a specific cluster of somatic symptoms (*b* = 4.03 years per unit increase on the average sum score, *p* < 0.0001). Brain-PAD estimates (in years) were residualized for age, sex, education level (years) and two dummy variables for scan location.

Using diagnostic status as a dichotomous between-group predictor we found that patients exhibited +1.75 years higher brain PAD than controls, but this difference did not reach statistical significance (Cohen’s *d* = 0.24). Within the patient group only, we found no significant associations with the age of onset of illness or duration of symptoms of either MDD or anxiety. A linear regression with brain PAD as the outcome and antidepressant use as predictor, showed that brain-PAD was significantly lower in antidepressants (AD) using patients compared with AD-free patients (*b* = −2.58 years, *p* = 0.01), but not control subjects (*b* = 0.59 years, *p* = 0.65) (Fig. 2A), while controlling for all covariates. Given the significant difference in brain-PAD between AD-free and AD-using patients, we included AD status as an additional covariate when comparing controls with the patient group, resulting in significantly higher brain-PAD in patients (+2.63 years [SE 1.10 years], Cohen’s *d* = 0.34, 95% CI 0.06–0.62, *p*_FDR_ = 0.048). We also added AD status as an additional covariate in a model to compare controls against specific MDD, anxiety, or comorbid patient groups (the proportion of subjects using AD in specific diagnostic groups was marginally different, Χ_(2)_=5.91, *p* = 0.052). This revealed significantly higher brain PAD in MDD (+2.78 years, Cohen’s *d* = 0.25, 95% CI −0.10–0.60, *p*_FDR_ = 0.048) and anxiety patients (+2.91 years, Cohen’s *d* = 0.27, 95% CI −0.08–0.61, *p*_FDR_ = 0.048), and a similar effect in the comorbid MDD and anxiety group (+2.23 years, Cohen’s *d* = 0.21, 95% CI 0.10–0.53) although only marginally significant (*p*_FDR_=0.08) (Table 2). There were no post hoc differences in brain PAD corrected for AD use between specific patient groups (MDD vs. anxiety vs. comorbid patients; P’s >0.46, Cohen’s *d*’s <0.07).

**Table 2:** Higher brain-PAD in depression and anxiety with correction for antidepressant use.

Ref	Predictor	*b* (years)	SE	t value	*P*	*P*_FDR_	Cohen’s *d*	SE	95% CI
Controls	Any patient	2.63	1.10	2.39	**0.02**	**0.048**	0.34	0.14	0.06–0.62
	MDD	2.78	1.32	2.11	**0.04**	**0.048**	0.25	0.18	−0.09–0.6
	Anxiety	2.91	1.31	2.22	**0.03**	**0.048**	0.27	0.17	−0.08–0.61
	Comorbid MDD and anxiety	2.23	1.28	1.74	0.08	0.08	0.21	0.16	−0.11–0.53

Age, sex, education level (years) and two dummy variables for scan location were included in all models. Antidepressant status was additionally included as covariate. Bold *p*-values indicate significance at the *p* < 0.05 level.

To gain more insight into the differences in brain-PAD between AD-free and AD-using patients, we post hoc calculated a derived daily dose by dividing the AD mean daily dose by the daily dose recommended by the World Health Organization (also see [35]). Brain-PAD was not significantly negatively associated with a derived daily dose of antidepressants in *n* = 74 patients (*b* = −0.91 year, *p* = 0.50) (Supplementary Figure S1). Of note, we excluded three subjects from this analysis as these AD-using patients were using Venlafaxine at doses higher than 150 mg/day, acting as a dual serotonin and norepinephrine reuptake inhibitor rather than acting as a selective serotonin reuptake inhibitor (SSRI) only [36]. Based on the above findings, both diagnostic and AD status were included in the multivariable model to test unique brain-PAD contributions.

### Selection of significant associations with clinical variables in all participants

Using a dimensional approach based on symptoms rather than diagnosis, we found that higher brain PAD was associated with higher total depression (*b* = 0.07 years per unit change on the Inventory of Depressive Symptoms, *p* = 0.03) and anxiety severity scores (*b* = 0.11 years per unit change on the Beck’s Anxiety Inventory, *p* = 0.01) across all participants (Fig. 2B, C). No significant associations were found for the mood/cognition (*b* = 0.89 years per unit increase on the average sum score, *p* = 0.27, Fig. 2D) or immunometabolic symptom clusters of depression (*b* = 0.45 years per unit increase on the average sum score, *p* = 0.62, Fig. 2E), but higher brain-PAD was strongly associated with more somatic symptoms of depression (*b* = 4.03 years per unit increase on the average sum score, *p* < 0.0001, Fig. 2F). There were no significant associations between brain-PAD and childhood trauma exposure (*b* = 0.23 years per unit change on the childhood trauma index, *p* = 0.26) or recent negative life events (*b* = 0.35 year per negative life event, *p* = 0.39).

### Selection of significant associations with somatic health in all participants

Higher brain PAD was associated with both higher BMI (*b* = 0.23 years per kg/m^2^, *p* = 0.02), as well as the number of somatic diseases under medical treatment (*b* = 1.45 years per somatic disease, *p* = 0.03). However, the latter association became nonsignificant if those with >2 chronic diseases (*n* = 4) were truncated to two chronic diseases (*b* = 1.29 years per somatic disease, *p* = 0.08).

### No associations with lifestyle or biological stress variables

There were no significant associations with any of the lifestyle variables (smoking, alcohol, and physical activity) or biological stress variables (inflammatory markers, ANS, and HPA axis), although the association with cortisol awakening response was trending toward significance (*b* = −0.23 years per nmol/l, *p* = 0.06). An overview of the separate linear regressions can be found in Table 3. Only antidepressant use, duration of symptoms or illness onset of depression and anxiety were assessed within patients only. Of note, our primary aim was to select predictors to subsequently include in one multivariable model. To avoid missing potential unique contributors, we also included significant predictors uncorrected for multiple comparisons. However, if considered separately, only somatic depressive symptoms would survive multiple-comparison correction, *p*_FDR_ = 0.003, with other predictors becoming nonsignificant.

**Table 3:** Overview of the brain-PAD associations with predictors of Interest.

Assessment	Predictor	*b* (years)	SE	*t* value	*P*	*P*_FDR_
*Clinical*	Depressive symptom severity	0.07	0.03	2.16	**0.03**	0.16
	Anxiety symptom severity	0.11	0.04	2.50	**0.01**	0.11
	Mood/cognition symptoms	0.89	0.81	1.10	0.27	0.50
	Somatic depression symptoms	4.03	1.04	3.87	**<0.00001**	**0.003**
	Immunometabolic symptoms	0.45	0.92	0.49	0.62	0.73
	Childhood trauma index	0.23	0.20	1.13	0.26	0.50
	Negative life events	0.35	0.41	0.86	0.39	0.63
*Within patients*	Antidepressant use	−2.58	1.02	−2.54	**0.01**	0.11
	Duration of depressive symptoms (proportion of time in the past 4 years)	−0.20	1.97	−0.10	0.92	0.95
	Duration of anxiety symptoms (proportion of time in the past 4 years)	−0.88	1.55	−0.56	0.57	0.73
	Age of onset of depression (years)	0.04	0.06	0.61	0.55	0.73
	Age of onset of anxiety (years)	0.01	0.05	0.09	0.93	0.95
*Somatic health*	BMI (kg/m^2^)	0.23	0.10	2.31	**0.02**	0.14
	Number of somatic diseases	1.29	0.72	1.79	0.08	0.30
*Lifestyle*	Alcohol (mean drinks per week)	−0.09	0.07	−1.33	0.19	0.49
	Smoking (cigarettes per day)	−0.07	0.05	−1.26	0.21	0.50
	Physical exercise (MET-minutes)	−0.06	0.13	−0.48	0.63	0.73
*Inflammation*	CRP (mg/l)	0.57	0.76	0.75	0.46	0.70
	TNF-α (pg/ml)	0.10	1.65	0.06	0.95	0.95
	IL6 (pg/ml)	0.60	0.98	0.61	0.54	0.73
*ANS*	Resting HR (bpm)	0.08	0.05	1.51	0.13	0.42
	RSA (ms)	−0.01	0.02	−0.46	0.65	0.73
	PEP (ms)	−0.04	0.03	−1.45	0.15	0.43
*HPA-axis*	AUCi (nmol/l/hr)	−0.23	0.12	−1.89	0.06	0.26
	AUCg (nmol/l/hr)	−0.11	0.11	−0.99	0.33	0.57
	Evening (nmol/l)	0.33	0.29	1.15	0.26	0.50

Age, sex, education level (years) and two dummy variables for scan location were included in all models. BMI Body Mass Index, MET-minutes Metabolic Equivalents, CRP C-reactive protein, TNF-α Tumor Necrosis Factor-α, IL6 Interleukin-6, ANS autonomic nervous system, HR heart rate, RSA respiratory sinus arrhythmia, PEP pre-ejection period, AUCi cortisol awakening response: area under the curve with respect to the increase, AUCg cortisol awakening response: area under the curve with respect to the ground, Evening Evening Cortisol. Bold *p*-values indicate significance at the *p* < 0.05 level.

### Multivariable model

The primary aim was to characterize the unique contributions of the selected significant predictors on the brain-PAD outcome. To this aim, we included diagnostic status (control vs. patient), MDD and anxiety-symptom scores, BMI, AD use, and the number of somatic diseases under treatment as predictors in a stepwise regression model with forward selection. Thus, predictors were successively added to an intercept-only model (Akaike’s Information Criterion [AIC] = 1115.81), only adding regression coefficients if they improved model fit (i.e., lower AIC). Using this method, we found that the best subset of variables to explain brain-PAD consisted of somatic depression symptoms and AD use (AIC = 1098.79). The results remained unchanged using a backwards elimination procedure. In sum, unique contributions to brain-PAD were observed for the somatic depression symptom cluster (*b* = 4.21 years per unit increase on average sum score, 95% CI 2.25–6.16, *p* < 0.0001) and AD use (*b* = −2.53 years, 95% CI −4.36–0.70, p = 0.007).

### Correlations with other biological clocks

With respect to the exploratory analyses with other biological aging indicators, we found low, nonsignificant (P’s > 0.13), correlations between brain-PAD, and three omics-based clocks (epigenetic, transcriptomic, and metabolomic) and telomere length (with Pearson *r* in the range of −0.03–0.15, Supplementary Figure S2). Brain-PAD was negatively associated with the proteomic clock (*r* = −0.24, *p* = 0.02), corrected for age.

## Discussion

The current study used a validated brain-age prediction model to show unique contributions of somatic depression symptom severity and antidepressant (AD) use to increased brain age (positive brain-PAD). AD users exhibited similar average brain PAD as control subjects, whereas those that were AD-free showed higher brain PAD. Correcting for AD use, we also showed that not only MDD patients, but also patients with anxiety disorder exhibited older-appearing brains compared with controls. Taken together, these findings may indicate the broad impact and heterogeneity of depression and anxiety disorders, as we illustrated that higher brain PAD was more selectively observed among persons with high somatic depression symptom burden. Surprisingly, there were no significant associations with lifestyle or biological stress systems.

To the best of our knowledge, we are the first to report advanced brain aging in anxiety disorders (i.e., generalized anxiety disorder, panic disorder, and social anxiety disorder) with an estimated +2.91 years on average, compared with controls, when correcting for AD use. This is consistent with the literature describing comparable effect sizes with respect to structural brain alterations in social anxiety disorder (Cohen’s *d* = 0.20) [37], and other anxiety-related disorders such as post-traumatic stress disorder (PTSD) (Cohen’s *d* = −0.17) [38], with PTSD patients also showing advanced brain PAD without correction for AD [17]. This observation may potentially offer an explanation as to why clinical anxiety is associated with an increased risk of dementia, even independent from depression [39], although further evidence is needed. The lack of any significant post hoc differences between specific diagnostic groups can likely be explained due to, among others, the high genetic correlation between the disorders [40], shared environmental risks, and overlapping personality traits of patients with depression and anxiety disorders [41]. Together with the transdiagnostic associations, this indicates that the brain-PAD metric is not sensitive to either depression or anxiety alone, but rather a general indicator that is impacted by mental and somatic health.

The most clinically relevant finding was that AD-using patients showed a similar brain age to controls, but not to AD-free patients, irrespective of specific depressive or anxiety disorder. This finding was previously overlooked in consortium data, presumably due to a lack of more detailed information on lifetime use, dosage, and duration of use of AD [16], highlighting the complementary values of well-characterized local samples and large-scale consortia. The AD finding was particularly interesting as the AD-using patients constituted a more severely depressed and anxious group as indicated by higher symptom severities compared with AD-free patients, potentially suggesting compensatory or normalizing mechanisms of AD, at least on the brain-PAD metric. This accords with earlier work reporting brain-PAD associations with therapeutic drugs, suggesting neuroprotective effects of lithium treatment in bipolar disorder patients (vs. no lithium) [42] and ibuprofen (vs. placebo) in healthy participants in an exploratory randomized controlled trial [43]. Yet, it remains unclear if and to what extent the brain-age-protective mechanisms overlap with, for example, increased neural progenitor cells [44], brain derived neurotrophic factor (BDNF) [45], or other serotonergic neuroplasticity processes implicated in AD use [46], or, alternatively, whether neuropharmacology affects the MRI signal [47]. Brain PAD was not positively associated with the duration of symptoms (either MDD or anxiety), suggesting that the AD effect was not driven by the duration of the disease and did not seem to be progressive. Taken together, these findings may suggest an age-related neuroprotective effect of AD, but interpretative caution is warranted as the current study was cross-sectional in nature and the dose–response association with AD not statistically significant. We also did not find associations with physical activity, while a previous study found an association between brain PAD and the daily number of flights of stairs climbed [48]. Future clinical interventions are needed to examine the short- and long-term effects of antidepressants and physical activity on biological aging, and potential differential and interaction effects of depression and anxiety disorders, an objective currently pursued by the MOod Treatment with Antidepressants or Running (MOTAR) study [49].

There were no associations with the cumulative childhood trauma index or the number of recent negative life events, different from the impact that adverse childhood experience commonly has on other biological age indicators such as telomere length [50], or epigenetic aging [51], albeit with small effects. Future studies with larger samples may potentially be more sensitive in picking up associations between brain PAD and childhood trauma. However, taken together, the current study found that advanced brain aging was more associated with current disease states, likely related to current symptom severity, rather than the result of cumulative exposure (i.e., no association with childhood trauma history, age of onset of illness, and duration of symptoms) or traits.

Furthermore, Cole and colleagues (2020) found significant associations between brain-PAD and several biomedical (e.g., blood pressure, diabetes, and stroke) and lifestyle variables (e.g., smoking status, alcohol-intake frequency), but not BMI, in the UK Biobank [20], albeit with a different, multi-modal brain-age prediction model but in a much larger sample size (>14,000 subjects). Although the current findings with somatic health broadly support previously associated diabetes [52] and stroke findings in UK Biobank, as well as the null finding with respect to physical activity, we did not identify associations with smoking or alcohol behavior [52]. More work is needed in terms of identifying unique or shared robust contributors to the brain-PAD metric, converging evidence across and between datasets, processing methods, and populations. Furthermore, each increase of one of the average sum score (range 0.80–4.00) of somatic depression symptoms, was associated with +4.20 years of added brain aging, independent from AD use. The somatic symptom cluster studied here consisted of items tapping into sleep, psychomotor, and other bodily symptom problems (see Supplement for all individual items within each cluster). This emphasizes the need to prevent and improve both mental and somatic conditions to promote healthy brain aging in psychiatric populations.

Surprisingly, none of the biological stress systems considered in the current study were predictive of brain aging, despite the strong association between brain PAD and somatic symptoms. This suggests that the biological dysregulations that commonly link depression to somatic health [5], were not directly contributing to advanced brain aging. On the other hand, it might indicate that the brain-PAD metric is more responsive to psychological stressors, rather than biological stressors. Other explanations might also be possible, as the current sample was relatively young (mean age ~40 years) and prior associations between, for example, brain aging and TNF-α, were found in a much older sample (mean age ~65 years) of which more than half also constituted patients with type two diabetes mellitus [52]. Longer follow-up duration of the current sample into old age and higher incidence of chronic disease may reveal associations between brain aging and biological stressors in the future. With respect to the inflammatory markers, it might be possible that blood levels of inflammatory markers do not accurately mirror central neuroimmune levels, although there is some evidence that C-reactive protein (CRP) measured peripherally also reflects central inflammation, at least in MDD [53]. Alternatively, a different potential biological mechanism that may explain the observed advanced brain aging in depression and anxiety disorders is metabolic dysregulation. Future studies could characterize the brain-PAD metric in more detail with respect to metabolic factors (e.g., blood pressure, triglycerides, and cholesterol), as these are well-established risk factors for unfavorable somatic conditions [54–57] and frequently co-occur with depression [58].

Only a handful of studies have compared multiple biological age indicators side-by-side [59–62], but the current findings support most work showing the very little overlap between biological clocks from different types of data [9]. However, the small but significant negative correlation between brain and proteomic aging suggests that a further study with more focus on the interplay between this peripheral and central proxy of aging is needed. Aging remains a multifaceted and complex process that may manifest differently across multiple biological levels and tissues.

### Limitations

It is important to mention that our sample had low statistical power to detect (some of) the relatively small-effect sizes in the current study, and only the association between brain PAD and somatic depressive symptoms would survive multiple-comparison correction if considered separately. At present, the large within-group variance of brain-PAD lacks utilitarian validity in a clinical context. We, therefore, emphasize the need for both methodological (i.e., brain-age models) and epidemiological replication (i.e., other and larger samples) to test the robustness of the effects. Another limitation is reflected by the lack of insights into the causal pathways implicated in advanced brain aging, given the cross-sectional nature of the study. However, a major strength is that we used a pre-established reference curve for healthy brain aging that has further potential for benchmarking, as the ENIGMA MDD working group encourages local research samples like ours to examine more detailed phenotypes that were not available within the consortium. Also important to note is that the effects of multivariate brain aging patterns (Cohen’s *d* = 0.34, between controls and all patients) were higher or comparable to other biological aging indicators (e.g., telomere length [Cohen’s *d* = 0.12] [63], epigenetic aging [*d* = 0.14]) [64], biological markers (e.g., BDNF [*d* = 0.23] [65], cortisol [*d* = 0.15–0.25] [66], and CRP [*d* = 0.15] [67]), and, most importantly, neuroimaging markers (e.g., hippocampal volume [*d* = −0.14] [68]), in other or (partly) overlapping samples.

## Conclusion

In summary, advanced brain aging in patients with MDD and anxiety seems to be most strongly associated with somatic depressive symptomatology. We also revealed that antidepressant medication use was associated with lower brain PAD, potentially suggesting that its use may have a protective effect on the age-related structural gray matter alterations observed in patients with MDD and anxiety, an effect previously overlooked in consortium data. Our results, therefore, emphasize the importance and complementary value of smaller, yet more homogeneous, datasets with harmonized data collection and well-characterized clinical phenotyping, compared with the large-scale consortium data needed for statistical power. Randomized clinical trials are needed to confirm whether advanced brain aging can be halted or reversed, by intervening on the cross-sectional somatic health indicators identified here, in pursuit of the characterization of a complex multifaceted process such as brain aging.

## Supplementary information

supplementary material
