# Supplementary material for: Contributing factors to advanced brain aging in depression and anxiety disorders
Source: Transl Psychiatry. 2021 Jul 21;11:402. doi: 10.1038/s41398-021-01524-2 (PMC8295382; doi:10.1038/s41398-021-01524-2)
Supplement: Supplementary file 1 — supplementary material [file 41398_2021_1524_MOESM1_ESM.docx]

**SUPPLEMENTARY MATERIAL**

**Contributing Factors to Advanced Brain Aging in Depression and Anxiety Disorders**

**SUPPLEMENTARY METHODS**

**NESDA MRI substudy in- and exclusion criteria**

The inclusion criteria for the MRI substudy were a DSM-IV diagnosis of major depressive disorder (MDD) and/or anxiety disorder (social anxiety disorder, panic disorder, generalized anxiety disorder) with a six month recency, but no other axis-I disorder. Control subjects were not allowed to have a history of any DSM-IV axis-I disorder. Exclusion criteria were a history of drug or alcohol abuse for both patients and controls, general MRI contraindications, and presence or history of a severe internal or neurological disorder. Additional exclusion criteria were the use of psychotropic medication with the exception of stable use of selective serotonin reuptake inhibitors (SSRIs) or infrequent benzodiazepine use for patients and use of any psychoactive medication for control subjects.

**Clinical assessments**

The Beck Anxiety Inventory (BAI) was used to measure the severity of anxiety symptoms as common in panic and generalized anxiety disorders, ranging from 0 (minimal) to 63 (severe)[[1]](https://paperpile.com/c/Dqd8HO/iPTSm). Depressive symptomatology was assessed with the Inventory Depressive Symptomatology (IDS)[[2]](https://paperpile.com/c/Dqd8HO/rcvCa). This 30-item questionnaire assesses the presence of all symptom domains of a major depressive episode in the past seven days on a 0-3 scale (not severe – severe), resulting in a total IDS score ranging from 0 (normal) to 84 (very severe). For both depression disorders and anxiety disorders, measures on duration (course) of psychopathology were assessed. The Life Chart Interview was used to determine the proportion of time in which symptoms relevant for the disorder were experienced in the past four years [[3]](https://paperpile.com/c/Dqd8HO/CfFBh). Childhood trauma before the age of 16 was assessed using the NEMESIS childhood trauma interview with personal history questions including a structured inventory of trauma exposure during childhood (emotional neglect, psychological abuse, physical abuse, sexual abuse, and important life-events in early life). In line with earlier work, a cumulative childhood trauma index (CTI) was created that reported the sum of the categories that were scored from 0 to 2 (0: never happened, 1: sometimes, 2: happened regularly), resulting in an index score ranging from 0-8 [[4]](https://paperpile.com/c/Dqd8HO/uhJlK). Frequent use of antidepressants (>50% of the time) was assessed through container inspection and categorized using World Health Organization Anatomical Therapeutic Chemical (ATC) classifications: selective serotonin reuptake inhibitors (ATC code N06AB), and other antidepressants (ATC codes N06AF, N06AG, N06AX).

**Depressive symptom clusters**

Previous studies based on the Netherlands Study of Depression and Anxiety (NESDA) and other populations have distinguished several different clusters of symptoms within the Inventory of Depressive Symptoms (IDS) [[2, 5, 6]](https://paperpile.com/c/Dqd8HO/rcvCa+X8hEF+7ixdW). Here, we largely follow the two factors identified by Wardenaar et al. 2010 [[7]](https://paperpile.com/c/Dqd8HO/lzR7E), but add a separate factor for immuno-metabolic features, thus distinguishing three symptom clusters: 1) mood/cognition symptom cluster, 2) immunometabolic symptom cluster, and 3) somatic symptom cluster (**Supplementary Table S1**). Given that the three different clusters consist of different numbers of items, we divided the total score of each cluster by the number of items in that cluster (mood/cognition: 15 items vs. immuno-metabolic: 5 items vs. somatic: 10 items) to obtain an average summary score.

**Supplementary Table S1: Categorization of Individual Items of the Inventory of Depressive Symptoms (IDS) into Three Symptom Clusters.**

| **Symptoms (IDS)** | **Depression symptom profiles** |
| --- | --- |
| Problems falling asleep | Somatic |
| Sleep during the night | Somatic |
| Waking-up too early | Somatic |
| Sleeping too much | Immuno-metabolic |
| Feeling sad | Mood/cognition |
| Feeling irritable | Mood/cognition |
| Feeling anxious or tense | Mood/cognition |
| Response of mood to good or desired events | Mood/cognition |
| Mood in relation to time of day | Mood/cognition |
| Quality of mood | Mood/cognition |
| Decreased appetite | Somatic |
| Increased appetite | Immuno-metabolic |
| Decreased weight | Somatic |
| Increased weight | Immuno-metabolic |
| Concentration/decision making | Mood/cognition |
| View of self | Mood/cognition |
| View of future | Mood/cognition |
| Thoughts of death/suicide | Mood/cognition |
| General interest | Mood/cognition |
| Energy level | Immuno-metabolic |
| Capacity for pleasure or enjoyment (excl. sex) | Mood/cognition |
| Interest in sex | Mood/cognition |
| Psychomotor retardation | Somatic |
| Psychomotor agitation | Somatic |
| Aches and pains | Somatic |
| Other bodily symptoms | Somatic |
| Panic/phobic symptoms | Mood/cognition |
| Constipation/diarrhea | Somatic |
| Interpersonal sensitivity | Mood/cognition |
| Leaden-paralysis/physical energy | Immuno-metabolic |

**Biological stress assessments**

*Inflammation*

A previous study also described the assessment of inflammation markers [[8]](https://paperpile.com/c/Dqd8HO/9xL71). Circulating plasma levels of C-Reactive Protein (CRP) (N=280), tumor necrosis level-α (TNF-α) (N=279), and Interleukin-6 (IL-6) (N=280) were assessed in duplicate. First, to measure plasma levels of CRP, an in-house enzyme-linked immunosorbent assay (ELISA) based on purified protein and polyclonal anti-CRP antibodies was used (Dako, Glostrup, Denmark). Intra- and inter-assay coefficients of variation were 5% and 10%, respectively. Second, plasma TNF-α levels were assessed using a high-sensitivity solid phase ELISA (Quantikine HS Human TNF-α Immunoassay, R&D systems, Minneapolis, MN, USA). Intra- and inter-assay coefficients of variation were 10% and 15% respectively. Finally, to measure plasma IL-6 levels a high sensitivity ELISA was used (PeliKine Compact, ELISA, Sanquin, Amsterdam, The Netherlands). Intra- and inter-assay coefficients of variation were 8% and 12%, respectively.

*Hypothalamic Pituitary Adrenal-axis*

The assessment of hypothalamic pituitary adrenal (HPA)-axis measures have also been previously described [[9]](https://paperpile.com/c/Dqd8HO/6flfn). To reliably assess the active, unbound form of cortisol participants were instructed to collect saliva samples at home (with minimal intrusiveness) on a regular (preferably working) day [[10]](https://paperpile.com/c/Dqd8HO/krN3Y). Salivettes were used to obtain saliva samples (Sarstedt, Nümbrecht, Germany) at six time points during a regular (work) day: at awakening (T1) and 30 (T2), 45 (T3), and 60 (T4) minutes later and at 10 PM (T5) and 11 PM (T6). The samples were stored in refrigerators and then returned by regular mail. After arrival, salivettes were centrifuged at 2000 × g for 10 min, aliquoted and stored at −80°C. Analyses of the cortisol were performed by competitive electrochemiluminescence immunoassay (Roche, Basel, Switzerland) [[11]](https://paperpile.com/c/Dqd8HO/eqqw5). The detection limit was 2.0 nmol/l and the intra- and inter-assay coefficients of variation were <10% [[12]](https://paperpile.com/c/Dqd8HO/zZC9j). Since the two evening values were highly correlated (r=0.75, p<.001), we averaged these two values.

*Autonomic Nervous System*

Subjects wore a so-called VU University ambulatory monitoring system (VU-ams) [[13]](https://paperpile.com/c/Dqd8HO/8Ehp8) during their interview. The VU-ams is a light-weight, unobtrusive device that records an electrocardiogram (ECG) and changes in thorax impedance (dZ) through 6 surface electrodes placed on the chest and the back [[14]](https://paperpile.com/c/Dqd8HO/m3Q5t). The heart rate was obtained by extracting the inter-beat interval time series from the ECG signal. Respiratory sinus arrhythmia (RSA) and pre-ejection period (PEP) were extracted from the combined dZ and ECG signals [[13]](https://paperpile.com/c/Dqd8HO/8Ehp8). RSA is a measure of cardiac parasympathetic (vagal) control, with high RSA levels reflecting high cardiac vagal control. We subtracted the shortest inter-beat interval during heart rate acceleration in the inspirational phase from the longest interbeat interval during deceleration in the expirational phase for all breaths to obtain a measure of RSA [[15]](https://paperpile.com/c/Dqd8HO/XPpJo). PEP is a measure of cardiac sympathetic control, as it can reliably index b-adrenergic inotropic drive to the left ventricle. Long PEP reflects low cardiac sympathetic control. PEP was defined as the interval from the beginning of the left ventricular electrical activity (ECG Q-wave onset), to the beginning of left ventricular ejection (B point in the dZ/dt signal) [[16]](https://paperpile.com/c/Dqd8HO/tDnL5). Given the fact that postural changes unrelated to autonomic activity affect PEP and RSA, data from periods in which participants were non-stationary (~15 min) were excluded [[17]](https://paperpile.com/c/Dqd8HO/BN0Wm). Movement was registered through vertical accelerometry. Automated scoring of RSA and PEP was checked by visual inspection, and valid data was averaged over 98.0 ± 24 (mean ± SD) min to create single HR, PEP and RSA values.

**Derived daily dose of antidepressants**

We had information available on daily dose of antidepressants (n=74), with 82% of the antidepressant (AD) users using serotonin and norepinephrine reuptake inhibitors (SSRIs) and the remaining 18% using Venlafaxine on doses <150 mg/day. Of note, we excluded three subjects because they were using Venlafaxine at doses higher than 150 mg/day, acting as a dual serotonin and norepinephrine reuptake inhibitor rather than acting as a Selective Serotonin Reuptake Inhibitor (SSRI) only [[18]](https://paperpile.com/c/Dqd8HO/qiNA8). The derived daily dose was calculated by dividing the AD mean daily dose by the daily dose recommended by the World Health Organization [[19]](https://paperpile.com/c/Dqd8HO/a7HiN) (also see [[20]](https://paperpile.com/c/Dqd8HO/56WdW)). However, brain-PAD was not significantly negatively associated with a derived daily dose of AD in (b=-0.91 year per g of AD per day, p=0.50) (**Supplementary Figure S1**).

**
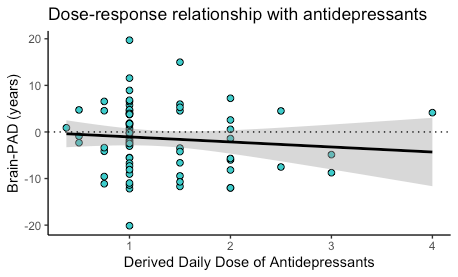
**

**Supplementary Figure S1. Dose-response relationship between brain-PAD and antidepressants.** Within the patient group, lower brain-PAD was not significantly associated with higher derived daily doses of antidepressants (AD) (SSRIs and Venlafaxine <1.5 g/day) (b=-0.94 years per g/day, p=0.50). Brain-PAD estimates (in years) were residualized for age, sex, education level (years) and two dummy variables for scanlocation.

**Other biological clocks**

To examine whether multivariate brain aging patterns were synchronized with telomere length (TL), and multivariate aging patterns from four omics-levels (epigenomics, proteomics, transcriptomics, metabolomics) we computed a correlation matrix between brain-PAD and these other five biological clocks. For a more detailed description of the biological clocks, please see [[21]](https://paperpile.com/c/Dqd8HO/mVimI). Briefly, ridge regression was used to predict the chronological age using data from different molecular levels. Chronological age effects were regressed out of all biological age predictions to indicate biological aging. Positive correlations indicated concordant biological aging processes, whereas negative intercorrelations indicated discordant aging patterns. There was low, non-significant, agreement between brain age and four other biological age indicators. However, we found a weak but significant inverse correlation between brain age and proteomic age, while controlling for age (**Supplementary Figure S2**).

**
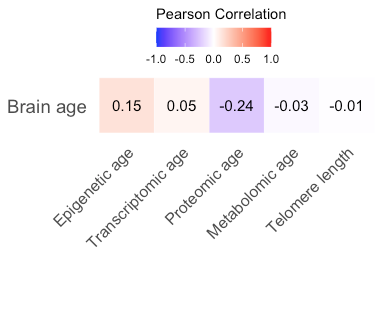
**

**Supplementary Figure S2. Correlation coefficients between brain-PAD and five other biological age indicators while controlling for age.** Similar methods were deployed predicting age from epigenetic, transcriptomic, proteomic, and metabolomic data. Telomere length was also included as a biological age indicator. Brain-PAD was not significantly correlated with either of the other biological age indicators, except for the proteomic clock (r=-0.24, p=0.02).

**REFERENCES**

1. [Beck AT, Epstein N, Brown G, Steer RA. An inventory for measuring clinical anxiety: psychometric properties. J Consult Clin Psychol. 1988;56:893–897.](http://paperpile.com/b/Dqd8HO/iPTSm)

2. [Rush AJ, Gullion CM, Basco MR, Jarrett RB, Trivedi MH. The Inventory of Depressive Symptomatology (IDS): psychometric properties. Psychol Med. 1996;26:477–486.](http://paperpile.com/b/Dqd8HO/rcvCa)

3. [Lyketsos CG, Nestadt G, Cwi J, Heithoff K, Et al. The Life Chart Interview: A standardized method to describe the course of psychopathology. Int J Methods Psychiatr Res. 1994;4:143–155.](http://paperpile.com/b/Dqd8HO/CfFBh)

4. [Hovens JGFM et al. Childhood life events and childhood trauma in adult patients with depressive, anxiety and comorbid disorders vs. controls. Acta Psychiatr Scand. 2010;122:66–74.](http://paperpile.com/b/Dqd8HO/uhJlK)

5. [Schaakxs R et al. Associations between age and the course of major depressive disorder: a 2-year longitudinal cohort study. Lancet Psychiatry. 2018;5:581–590.](http://paperpile.com/b/Dqd8HO/X8hEF)

6. [Hegeman JM et al. The subscale structure of the Inventory of Depressive Symptomatology Self Report (IDS-SR) in older persons. J Psychiatr Res. 2012;46:1383–1388.](http://paperpile.com/b/Dqd8HO/7ixdW)

7. [Wardenaar KJ et al. The structure and dimensionality of the Inventory of Depressive Symptomatology Self Report (IDS-SR) in patients with depressive disorders and healthy controls. J Affect Disord. 2010;125:146–154.](http://paperpile.com/b/Dqd8HO/lzR7E)

8. [Vogelzangs N et al. Association of depressive disorders, depression characteristics and antidepressant medication with inflammation. Transl Psychiatry. 2012;2:e79.](http://paperpile.com/b/Dqd8HO/9xL71)

9. [Vreeburg SA et al. Major depressive disorder and hypothalamic-pituitary-adrenal axis activity: results from a large cohort study. Arch Gen Psychiatry. 2009;66:617–626.](http://paperpile.com/b/Dqd8HO/6flfn)

10. [Kirschbaum C, Hellhammer DH. Salivary cortisol in psychoneuroendocrine research: Recent developments and applications. Psychoneuroendocrinology. 1994;19:313–333.](http://paperpile.com/b/Dqd8HO/krN3Y)

11. [van Aken MO, Romijn JA, Miltenburg JA, Lentjes EGWM. Automated measurement of salivary cortisol. Clin Chem. 2003;49:1408–1409.](http://paperpile.com/b/Dqd8HO/eqqw5)

12. [Pruessner JC, Kirschbaum C, Meinlschmid G, Hellhammer DH. Two formulas for computation of the area under the curve represent measures of total hormone concentration versus time-dependent change. Psychoneuroendocrinology. 2003;28:916–931.](http://paperpile.com/b/Dqd8HO/zZC9j)

13. [Licht CMM et al. Increased sympathetic and decreased parasympathetic activity rather than changes in hypothalamic-pituitary-adrenal axis activity is associated with metabolic abnormalities. J Clin Endocrinol Metab. 2010;95:2458–2466.](http://paperpile.com/b/Dqd8HO/8Ehp8)

14. [Willemsen GH, De Geus EJ, Klaver CH, Van Doornen LJ, Carroll D. Ambulatory monitoring of the impedance cardiogram. Psychophysiology. 1996;33:184–193.](http://paperpile.com/b/Dqd8HO/m3Q5t)

15. Electrophysiology, Task Force of the European Society of Cardiology the North American Society of Pacing [and Electrophysiology. Heart rate variability: standards of measurement, physiological interpretation and clinical use. Circulation. 1996;93:1043–1065.](http://paperpile.com/b/Dqd8HO/XPpJo)

16. [Berntson GG et al. Autonomic cardiac control. III. Psychological stress and cardiac response in autonomic space as revealed by pharmacological blockades. Psychophysiology. 1994;31:599–608.](http://paperpile.com/b/Dqd8HO/tDnL5)

17. [Houtveen JH, Groot PFC, De Geus EJC. Effects of variation in posture and respiration on RSA and pre-ejection period. Psychophysiology. 2005;42:713–719.](http://paperpile.com/b/Dqd8HO/BN0Wm)

18. [Debonnel G et al. Differential physiological effects of a low dose and high doses of venlafaxine in major depression. Int J Neuropsychopharmacol. 2007;10:51–61.](http://paperpile.com/b/Dqd8HO/qiNA8)

19. [Anatomical Therapeutic Chemical (ATC) Classification Index: Alphabetically Sorted According to Nonproprietary Drug Name ; Only ATC 5th Levels are Included. WHO Collaborating Centre for Drug Statistics and Methodology.](http://paperpile.com/b/Dqd8HO/a7HiN)

20. [Licht CMM et al. Association between major depressive disorder and heart rate variability in the Netherlands Study of Depression and Anxiety (NESDA). Arch Gen Psychiatry. 2008;65:1358–1367.](http://paperpile.com/b/Dqd8HO/56WdW)

21. [Jansen R, Han LKM et al. An integrative study of five biological clocks in somatic and mental health. eLife. 202](http://paperpile.com/b/Dqd8HO/mVimI)1[.](http://paperpile.com/b/Dqd8HO/mVimI)
